# Screening for personality disorder with the Standardised Assessment of Personality: Abbreviated Scale (SAPAS): further evidence of concurrent validity

**DOI:** 10.1186/1471-244X-10-10

**Published:** 2010-01-28

**Authors:** Morten Hesse, Paul Moran

**Affiliations:** 1 Centre for Alcohol and Drug Research, University of Aarhus, Denmark; 2 Health Services and Population Research Department, Institute of Psychiatry, King's College London, UK

## Abstract

**Background:**

The assessment of personality disorders (PD) is costly and time-consuming. There is a need for a brief screen for personality disorders that can be used in routine clinical settings and epidemiological surveys. Aims: To test the validity of the Standardised Assessment of Personality: Abbreviated Scale (SAPAS) as a screen for PD in a clinical sample of substance abusers.

**Methods:**

Convergent validity of the SAPAS with both categorical and dimensional representations of personality disorders was estimated.

**Results:**

In this sample, the SAPAS correlated well with dimensional representations of cluster A and C personality disorders, even after controlling for ADHD symptoms, anxiety/depression symptoms and recent substance use. The SAPAS was also significantly associated with total number of PD criteria, although correlation with categorical measures of PD was weak.

**Conclusions:**

The SAPAS is an valid brief screen for PD as assessed dimensionally.

## Background

Personality pathology is common among substance dependent patients [1]. Substance dependent patients display the full range of personality disorders, and these diverse disorders predict impairment in different areas of functioning [2]. A growing body of research suggests that a dual focus on both personality disorder and substance use disorder is superior to treatment that focuses only on the substance use disorder itself (e.g [3]).

The Structured Assessment of Personality Abbreviated Scale (SAPAS) is an eight-item screening interview for personality disorder [4]. Its purpose is to produce a dimensional score that represents the likelihood that a person has a personality disorder in general, rather than to screen for particular types of personality disorders or patterns. It produces a score that ranges from 0 to 8. In the original study with psychiatric patients, a score of 3 or more was both sensitive and specific as a measure of the presence of a personality disorder according to the Structured Clinical Interview for the DSM-IV- Axis II [4]. It was designed to be so brief that it could be used in both routine clinical assessment when pressed for time, and potentially in community surveys.

In a previous study of a Danish population, it was found that the SAPAS correlated with staff-ratings of externalizing behaviour and global assessment of functioning in a methadone maintenance clinic [6]. However, while the previous study assessed correlations with functioning, it did not assess convergent validity with other measures of personality disorders. The original study of the SAPAS tested the value of the instrument as a measure of personality disorder in a psychiatric sample, but did not test associations with other problem areas, such as functioning, symptoms of anxiety or depression, or attention deficit-hyperactivity disorder [4]. Thus, neither study has assessed the possibility that the SAPAS as a measure of personality pathology is confounded by other psychopathology.

There is an ongoing debate regarding the independence of axis II disorders from axis I disorders [5]. As axis II disorders share a range of properties with axis I disorders, such as impaired functioning, difficulty in decision making and subjective well-being, it is important that screening tools designed to study the presence of personality disorders have discriminant validity against axis I disorders. This has not yet been studied for the SAPAS in any language.

In addition, it is unclear how well the SAPAS measures the full range of personality pathology. The DSM-IV lists 10 different personality disorders (plus 3 appendix disorders for further study) [8], and the SAPAS may have greater salience for some specific areas of personality pathology

The aim of the present study was to examine the convergent validity of the SAPAS with structured interviews from both a categorical and a dimensional perspective, using a different sample than those in the original study.

Specifically, we wanted to study the following questions:

• Is the SAPAS associated with dimensional scores representing personality disorder in general, defined as the total number of axis II criteria met?

• Is this association robust across individual disorders, and the three clusters of the DSM-IV?

• Is this association robust after controlling for gender, age, current symptoms of anxiety and depression, symptoms of attention-deficit hyperactivity disorder, and recent substance use?

## Methods

### Procedure

We set out to examine the concurrent validity of a mini-interview for personality disorder Structured Assessment of personality - Abbreviated Scale [4]. In order to do this, we conducted a series of secondary analyses of data from a randomized controlled trial of personality disorder psychoeducation for substance use disorders. The subjects for this paper were patients randomized to the experimental condition in the study (n = 36), and 18 training cases. The 36 experimental cases were taken from consecutive admissions to the Centralized intake units where the randomized study took place. The 18 cases consisted of 10 cases who were admitted shortly before the trial began, plus 8 volunteers who were assessed for other purposes as part of their treatment for substance use disorders, who agreed to give consent for the use of their data for research purposes.

Subjects were approached by their caseworkers, and informed that they had the option to be assessed for personality disorders and other psychopathology as part of an ongoing study. Those who agreed were referred to a research technician, who explained the rationale of the study.

Subjects gave consent to participate in the study no earlier than 24 hours after being informed of the purpose of the study by the research technician. The subjects were told that the data collected for the study would be used for research purposes, and at the same time be used for their treatment. After assessment, subjects were first given an individual feedback about the results of the assessment, and then, if they expressed their interest in this, this feedback was passed on to their caseworker. Subjects were also informed that without their consent, no information from the interviews would be passed on to any third party.

The interviews were conducted on two different days. On the first day, the SAPAS, and all of the non-personality related instruments were administered, and on the second interview day, the AUDADIS, the PRISM and the NPI-16 were administered (see below for instrument descriptions).

### Sample description

The sample was 85% male, and the mean age was 32.5 (range: 19 to 54). Among the respondents, 74% had used alcohol in the last 30 days before interview, 66% had used cannabis, 9% amphetamine, 30% cocaine, 11% heroin or other opiates, and 6% benzodiazepines. All patients in the sample scored 3 or more on the Severity of Dependence scale, indicating substance dependence. The mean score for the Kessler 6 was 10.8 (range: 0 to 21), indicating elevated scores on depression and anxiety. The mean score for the ADD scale was 19.7 (range 2 to 34), where scores above 23 indicate likely ADD. The mean for the hyperactivity scale was 17.0 (range: 7 to 34) where scores above 23 indicate likely hyperactivity disorder.

### Measures

The Structured Assessment of Personality Abbreviated Scale (SAPAS) is an eight-item screening interview for personality disorder [4]. Each item is worded as a question to be answered with yes or no (e.g., item 1: "In general, do you have difficulty making and keeping friends?"). When the response is given that indicates pathology (i.e., yes to item 1), the interviewer must follow up by asking if that is true in general. With eight yes/no questions followed by up to eight follow-up questions, the SAPAS will normally be completed in less than a minute. As the SAPAS is a set of indicators covering multiple areas, it is not designed to be unidimensional. Rather, the SAPAS is designed to cover different areas of personality.

The Kessler 6 (K6) is a 6-item interview. Each question starts with the expression "How often in the past month did you feel ..." and offers specific symptoms such as "restless or fidgety," "nervous," and "so depressed that nothing could cheer you up?" The 5 possible responses range from "none of the time" to "all of the time" and are scored from 1 to 5; the items are summed to obtain a total score. The K6 has been found to correlate substantially with both the Comprehensive International Diagnostic Interview-Short Form and the World Health Organization Disability Assessment Schedule [14]. Internal consistency alpha for the present sample was 0.79.

The Adult ADHD Self-Report Scale (ASRS) [15] is an 18-item self-report scale used to screen for adult symptoms of attention-deficit hyperactivity disorder. The ASRS has been found to possess excellent validity [15], including convergence with predicted genetic risk markers for ADHD [16]. For the present sample, the internal consistency alpha was 0.84 for attention deficit symptoms and 0.74 for hyperactivity symptoms.

The Psychiatric Research Interview for Substance and Mental Disorders (PRISM) interview is a semi-structured interview assessing a range of disorders that are commonly co-morbid with substance use disorders. Each item is assessed by asking a question, and when positive answers are given, follow-up questions are asked to assess the severity and persistence of a symptom. Each item is scored as 1 (absent), 2 (sub-threshold) or 3 (clinically significant). The PRISM has demonstrated validity for antisocial and borderline personality disorder (e.g [12]). In the present study, we assessed the interrater reliability of 27 taped interviews. For individual criteria for borderline personality disorder, the average interrater agreement assessed as intraclass correlation ranged from 0.66 to 0.98. For the number of criteria satisfied, the intraclass correlation was 0.93. For individual antisocial personality disorder criteria, the intraclass correlation for individual items ranged from 0.63 to 0.98, and the intraclass correlation for number of criteria satisfied was 0.98.

The Alcohol Use Disorder and Associated Disabilities interview Schedule-IV (AUDADIS-IV) is a fully structured interview covering a range of disorders, including personality disorders. At the time of this study, only the proportions of the interview that covered avoidant, dependent, obsessive-compulsive, paranoid, schizoid, histrionic and antisocial personality disorder were published. The AUDADIS has demonstrated validity and reliability [13], although it has rarely been used in clinical samples. The AUDADIS asks one or more questions for each criterion for Axis II, and given an affirmative answer, the interviewer must ask "Did this ever trouble you or cause problems at work or school, or with your family or other people?" Since these questions must be answered yes or no, there is no clinical judgement with regard to the interview, and interrater reliability of the recordings was not assessed. Internal consistency alpha based on tetrachoric correlations for the present sample ranged from 0.69 to 0.94 for the AUDADIS scales.

The Narcissistic Personality Inventory-16 (NPI-16) [12] is an abbreviated version of the Narcissistic Personality Inventory. The NPI uses a forced-choice format with a narcissistic and a non-narcissistic response for each item (e.g., "I am apt to show off if I get the chance" and "I try not to be a show off"). The 16-item version was developed to capture the different aspects of narcissism measured by the original NPI, and has excellent convergent validity with the original version, and good predictive validity [12]. Internal consistency alpha based on tetrachoric correlations for the NPI in this sample was 0.83.

### Analyses

For the patients (n = 54) who completed both the SAPAS and the full personality disorder assessment, we estimated the agreement between the SAPAS with the cut-off of 3 or more based on the original article [4]. We also correlated the Spearman rank-order correlations between the SAPAS and number of personality disorder criteria by cluster (excluding schizotypal and narcissistic personality disorder), and for each individual criterion. We report correlations of 0.1-0.3 as low, 0.3-0.5 as moderate, and correlations >0.5 as large, following Cohen [17].

We conducted a series of linear regressions to assess the association between the SAPAS and number of personality disorder criteria controlling for various confounders, one for each cluster, and one for total number of personality disorder criteria. In each regression, the dependent variable was symptom count for personality disorders (by cluster, or in total). The covariates were age, gender, and severity of anxiety or depression symptomatology as measured by the Kessler 6 interview, and severity of attention deficit problems and hyperactivity as measured by the ADHD Self-Report Scale.

In terms of number of criteria, we used the sums of the PRISM borderline and antisocial and AUDADIS histrionic personality for cluster B pathology, the sum of avoidant, dependent and obsessive-compulsive personality disorder criteria for cluster C pathology, and the sum of paranoid and schizoid personality disorder criteria for cluster A pathology.

The NPI is not a diagnostic instrument per se, and while it has been shown to predict important indicators of narcissistic pathology, we did not include the NPI as a part of cluster B pathology in the analyses.

### Ethics

Danish IBRs do not evaluate studies that do not involve invasive procedures or the manipulation of pharmacotherapy or diet. Dr. Peter Ege, senior consultant of social medicine in the City of Copenhagen, did an informal review of the ethical implications of the study.

## Results

### Categorical agreement with diagnostic interview

Among the 54 patients in the sample, the most common personality disorders were antisocial (PRISM, 52%), paranoid (AUDADIS, 44%), borderline (PRISM, 41%), and histrionic (AUDADIS, 37%) personality disorder.

Of the 54 patients who could be included in this analysis, 35 (65%) scored 3 or more on the SAPAS, and 49 (91%) received a diagnosis of at least one personality disorder based on either the PRISM (borderline or antisocial) or the AUDADIS interview (other personality disorders). The agreement was statistically significant (κ = 0.22, p = 0.02) although was weak.

### Dimensional agreement between number of SAPAS items endorsed and number of personality disorder criteria satisfied

The correlations between the SAPAS and the criteria count for each personality disorder and by cluster are summarized in table 1. Correlations varied substantially. Correlations between the SAPAS and paranoid and avoidant personality disorder features were large, and statistically significant. The correlations between the SAPAS and schizoid, dependent and borderline personality disorder were moderate.

**Table 1:** Rank-order correlations between personality disorder criteria counts and the SAPAS (N = 54) (based on AUDADIS except as indicated)

	Rho	Probability
Cluster A criteria	0.58	0.00

Paranoid	0.53	0.00

Schizoid	0.40	0.00

Cluster B criteria	0.39	0.00

Antisocial^1^	0.04	0.78

Histrionic	0.26	0.06

Borderline^2^	0.47	0.00

Cluster C criteria	0.59	0.00

Avoidant	0.55	0.00

Dependent	0.48	0.00

Obsessive-compulsive	0.25	0.06

Total number of personality disorder criteria^3^	0.61	0.00

NPI-16	-0.02	0.87

^1^PRISM-interview adult symptoms only.

^2^PRISM-interview.

^3^Across both AUDADIS and PRISM.

Correlations between the SAPAS and antisocial, histrionic and obsessive-compulsive personality disorder were non-significant and low. Also shown in table 1 is the Spearman correlation between the SAPAS and the NPI-16, also low (-0.02).

### Regression of SAPAS on criteria for personality disorders

After controlling for gender, age and symptoms of anxiety and depression as measured by the Kessler 6 interview [14], and hyperactivity and attention deficit disorder on the ADHD Self-Report Scale [15], the SAPAS remained significantly associated with total number of PD criteria (p = 0.03), and with number of cluster A criteria (p = 0.003) and cluster C criteria (p = 0.01), but not cluster B criteria (p = 0.95) (see table 2). In the multivariate analyses, cluster A criteria were additionally associated with attention disorder (p = 0.02), cluster B criteria were associated only with hyperactivity severity (p = 0.006), and cluster C criteria were additionally associated with symptoms of anxiety and depression (p = 0.03), and low degree of substance use (p = 0.03).

**Table 2:** Multivariate associations between SAPAS scores and number of PD criteria by cluster (N = 52)

	A (odd-eccentric)		B (dramatic-erratic)		C (anxious-fearful)		Total PD	
	**Beta**	**T(44)**	**Beta**	**T(44)**	**Beta**	**T(44)**	**Beta**	**T(44)**

SAPAS	0.42	**3.20	0.01	0.06	0.28	*2.54	0.26	*2.24

K6	0.02	0.15	0.12	0.67	0.30	*2.29	0.19	1.38

ADD	0.40	*2.37	-0.04	-0.18	0.19	1.31	0.20	1.29

Hyperactivity	0.08	0.59	0.46	**2.87	0.14	1.19	0.29	*2.37

OTI drug and alcohol use	0.02	0.20	-0.12	-0.88	-0.21	*-2.24	-0.14	-1.35

Gender	-0.17	-1.43	0.16	1.16	0.13	1.28	0.07	0.68

Age	0.09	0.78	0.04	0.32	-0.04	-0.44	0.03	0.30

Total R^2^adjusted for degrees of freedom	0.42		0.20		0.59		0.53	

F(7,44)	***6.32		*2.79		***11.59		***9.25	

Notes: Beta values represent association between SAPAS and number of criteria for personality disorder in cluster. K6: Kessler 6. ADD: Attention Deficit Disorder score. * p < 0.05. ** p < 0.01.

## Discussion

As a dimensional measure of the construct of personality disorder, the SAPAS possesses several attractive properties: it correlates highly with the number of interview-based criteria for personality disorder, and this correlation remains significant even after controlling for gender, age, symptoms of anxiety and depression, attention-deficit hyperactivity disorder symptoms, and recent substance use. The associations between the SAPAS and cluster A and C disorders were also robust across all confounders tested.

However, it also has important limitations: the SAPAS does not cover the full range of personality disorders equally well. It does not correlate highly with antisocial, histrionic and obsessive-compulsive personality disorder, and with trait narcissism.

Some other correlates of personality disorder severity deserve comment. These other correlates of personality disorder criteria varied by cluster. Cluster A criteria (paranoid and schizoid) showed an independent association with attention disorder type symptoms. Previous research has shown an elevated risk of all types of personality disorder across types of personality disorders [17].

The current study also has important limitations. The focus on borderline and antisocial pathology meant that we chose an instrument that is different from the instrument used for the other disorders, the AUDADIS.

Hyperactivity type symptoms were significantly associated with cluster B disorders. Borderline and antisocial personality disorders are both believed to share a number of features with the full ADHD syndrome, and in particular with the hyperactivity part of ADHD [18]. Prospective research suggests that ADHD is commonly a precursor of borderline and antisocial personality disorders [19].

Some limitations must be acknowledged. This study is limited to substance abusers seeking outpatient treatment. However, given existing evidence from psychiatric patients and methadone maintenance treatment, the present study adds to our confidence in the validity of the instrument. The sample size is also a limitation of the present study. However, given the focus on convergent validity, weak to moderate correlations that are not robust to the influence of covariates is unacceptable.

## Conclusions

In summary, we found the SAPAS was an acceptable screen for the odd/eccentric and anxious/fearful dimensions of PD, although it performed less satisfactorily for the domain of dramatic/impulsive personality disturbance. It is likely that a dimensional classification system for personality disorder will be introduced in DSM-V [19] and in the light of this, the SAPAS could be of great value to both clinicians and researchers as a screen for personality disturbance.

## Conflicts of interests

The authors declare that they have no competing interests.

## Authors' contributions

MH organized the study. Both authors planned the current report, MH conducted the statistical analyses, and drafted the manuscript. PM reviewed the manuscript, and both authors revised the manuscript several times in conjunction.

## Pre-publication history

The pre-publication history for this paper can be accessed here:

http://www.biomedcentral.com/1471-244X/10/10/prepub
